# Surgical site infection and associated factors among women underwent cesarean delivery in Debretabor General Hospital, Northwest Ethiopia: hospital based cross sectional study

**DOI:** 10.1186/s12884-019-2442-0

**Published:** 2019-08-29

**Authors:** Mihretu Molla, Kiber Temesgen, Tewodros Seyoum, Mengstu Melkamu

**Affiliations:** Department of Midwifery College of Medicine and Health Sciences, University of Gondar, Gondar, Ethiopia

**Keywords:** Surgical site infection, Cesarean delivery, Debretabor General Hospital

## Abstract

**Background:**

Cesarean section rates have been increasing dramatically during the past three decades and surgical site infections are becoming a leading cause of morbidity and mortality among women undergoing cesarean deliveries. However there is lack of sound evidence on both the magnitude of the problem and the associated factors in developing countries including Ethiopia. The purpose of this study was to assess proportion of surgical site infection and associated factors among women undergoing cesarean delivery in Debretabor General Hospital.

**Methods:**

An institution based cross sectional study was conducted from May to December / 2017. All women delivered by cesarean section in Debretabor General Hospital during data collection period were our study population. Data were collected using Pre-tested, semi-structured questionnaire/ data extraction tool and post discharge phone follow up and analyzed using SPSS version 20. Logistic regression model was used to determine the association of independent variables with the outcome variable and odds ratios with 95% confidence interval were used to estimate the strength of the association.

**Results:**

Proportion of surgical site infection among cesarean deliveries was about 8% (95%Cl: 5.4, 11.6). Pregnancy induced hypertension (AOR = 4.75, 95%CI: 1.62, 13.92), chorioaminitis (AOR = 4.37, 95%CI: 1.53, 12.50), midline skin incision (AOR = 5.19, 95% CI: 1.87, 14.37 and post-operative hemoglobin less than 11 g/deciliter (AOR = 5.28, 95%CI: 1.97, 14.18) were significantly associated with surgical site infection.

**Conclusions:**

Pregnancy induced hypertension, chorioaminitis, midline skin incision and post-operative hemoglobin of less than 11 g/deciliter were independent factors associated with surgical site infection. Cesarean deliveries with concomitant pregnancy induced hypertension, chorioaminitis and post-operative anemia needs special care and follow up until surgical site infection is ruled out. It is also advisable to reduce generous midline skin incision and better replaced with pfannensteil incision.

## Background

Surgical site infection (SSI) is an infection occurring within 30 days after the operation and involves the skin and subcutaneous tissue and/or the deep soft tissue of the incision [1–3].

SSI is a leading cause of morbidity and mortality among women undergoing cesarean section (CS) with reported rates of 3–15% [4–6]. High rates of SSI following CS were reported in several lower and middle income countries: 16.2% in a study from Nigeria, 19% from Kenya, 10.9% from Tanzania and 9.7% from Viet Nam among others. [7, 8].

According to a study done in China, 3.34% of cesarean deliveries (CDs) were complicated by SSI [9]. In contrast,SSI complicated 12.6% [10] and 24.3% [11] of CDs at Dhulikhel Hospital (Nepal) and Karachi (Pakistan) respectively. A prevalence rate of 6.2% [12] SSI following CDs was reported in a study done in Ankara (Turkey) and lower rate of SSI (3.7%) was reported in study done in Israel [13]. A study at Teaching Hospital in Rwanda revealed that 4.9% CDs were complicated by SSI [14].

However, SSI following CDs was reported on almost half (48.2%) of CDs at Tanzanian Tertiary Hospital [15]. In Ethiopian context, the prevalence of SSI at Hawassa Teaching and Referral Hospital was 11% [16]. Meanwhile 9.4% of CDs were complicated by SSI at Assela Teaching and Referral Hospital [17]. Similarly study done at Lemlem karl General Hospital (Tigrai region) revealed 6.8% of CDs had developed SSI [18].

Pre-operative conditions such as prolonged labor, prolonged rupture of membrane (PROM), more than 5 digital vaginal examination, chorio-amnionitis, American society of Anesthesiologist (ASA) health status classification ≥3, fewer years of education, higher prior births, prior diagnosis of hypertension and Diabetes Mellitus (DM) were identified as significant factors for SSI following CDs [10, 15, 16, 19–22]. Similarly intra operative conditions like prolonged duration of surgery, wound contamination class III, vertical skin incision and interrupted skin suturing were associated factors for developing SSI [8, 9, 15, 16, 19, 23, 24]. CD for emergency conditions like non-reassuring fetal heart rate pattern (NRFHRP) was also significantly associated factor for developing SSI [19, 24, 25]. In addition, Postoperative anemia was also found to be a post-operative factor associated with SSI [16, 26].

In Ethiopian context post-partum infection is the fourth leading cause of maternal mortality and morbidity from which SSI shares the largest proportion [27]. Even if admissions following CDs due to SSI have been routine activities of health care institutions in our set up, there is lack of strong evidence on both the magnitude of the problem and the associated factors making prevention mechanisms less effective. Few efforts have been done in terms of both quantifying the problem and identifying the determinant factors.

A study conducted at Jimma University specialized hospital had reported the magnitude of then SSI on obstetric cases and had identified associated factors [28]. But the reported magnitude was not specific to SSI following CD and the identified factors were not tested for confounding effects. More over 8 years has been lapsed after this study and the tracing mechanism of study participants after discharge was not as such reliable. More recently three studies have been done in Hawassa, Assela and Maichew and reported their respective magnitude of SSI following CDs and the determinant factors. But all of them were conducted retrospectively using only secondary data. Therefore, this study aimed to quantify the proportions of post-caesarean wound infection and to identify the associated factors among cesarean deliveries (CDs) at Debretabor General Hospital (DGH). This will have a fundamental role in forming a basis for future studies and measures to prevent SSI following CDs at this hospital level in particular and nationwide in general.

## Methods

### Study design and period

Hospital based cross-sectional study was conducted from May 20 to December 15 /2017.

### Study setting

This study was conducted in DGH located in Debretabor town of Amhara Regional State. Debretabor town is about 98 km away to the East of Bahir-Dar (the capital of Amhara regional state) and about 667 km away to North of Addis Ababa (the capital of Ethiopia). DGH is the only general hospital in the south Gondar Zone having over 102 beds. Obstetrics and gynecology service is organized to a ward with a capacity of 22 beds and an operation room for emergency cesarean deliveries, maternal and child health unit, gynecology referral clinic and safe abortion clinic. The service is provided by two obstetrician and gynecologists, five integrated emergency surgical officers and over 22 professional and diploma midwives. About 3600 deliveries are conducted every year of which about 700 of them are by CS.

### Characteristics of participants

Women who delivered by CS from May 20 to December 15/2017 at DGH were included in this study. Women who didn’t have tracing mechanisms (if either the woman and/or her attendant/family didn’t have phone number) after discharge were excluded.

### Variables of the study

#### Outcome variable

SSI following CD (present/absent).

#### Independent variables/predictor variables include

Age, residence, educational status, marital status, body mass index (BMI), health status classification, hypertension, DM, heart disease, anemia, gravidity, parity, onset of labor, status of membrane,duration of ROM, duration of labor, number of vaginal examination, gestational diabetes mellitus (GDM), gestational age, antenatal care (ANC) follow up, chorioaminionitis, steroid therapy, pregnancy induced hypertension (PIH), type of CS, duration of the operation, skin incision type, skin suturing type, wound classification, estimated blood loss (EBL), intraoperative blood transfusion, and post-operative hemoglobin level.

### Enrolment of study participants

**Fig. 1:**
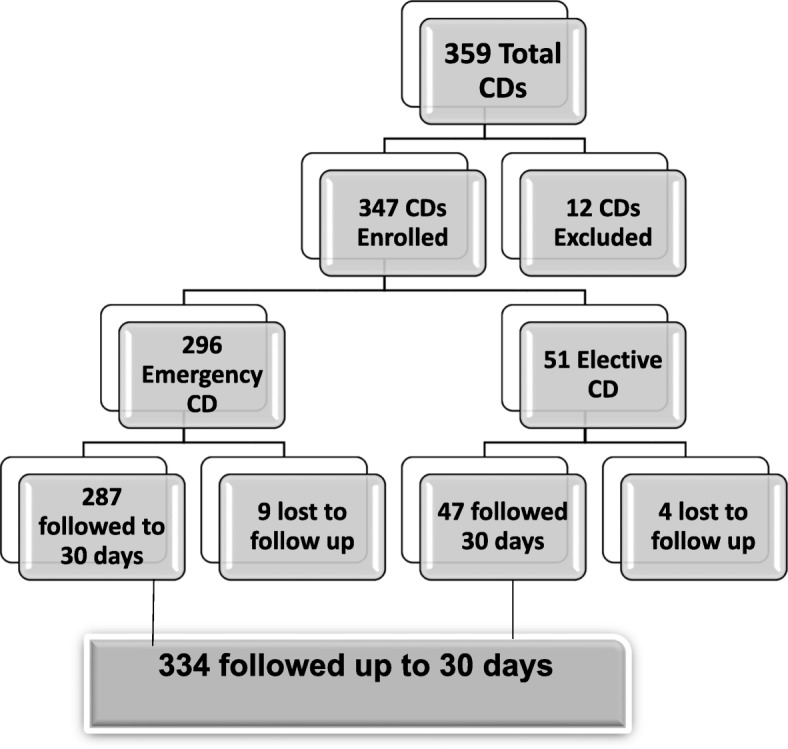
Schematic presentation of enrolment of study participants for surgical site infection and associated factors among cesarean deliveries at Debretabor general hospital, 2017

Census approach was used to include women delivered by CS from May 20 to December 15/2017. There were a total of 359 CDs of which 12 of them were excluded due to absence of tracing mechanism after discharge. The remaining 347 study participants were included in the study but 334 of them were traced for 30 days (Fig. 1).

For the sake of clarity, some of the operational definitions used in this research include: SSI was defined as per the criteria of the Centers for Disease Control and Prevention (CDC) guideline [1]. ASA physical status was defined as assessment of the woman’s preoperative physical condition using the ASA Classification of Physical status [29].

Wound class was defined as assessment of the degree of contamination of a surgical wound at the time of the operation by a person involved in the surgical procedure [29]. Steroid therapy was defined as preoperative maternal treatment with either betamethasone or dexamethasone for fetal lung maturity. Duration of membrane rupture was determined by the time lapsed in hour from either mother’s complain of leakage of liquor or artificial rupture of membrane to skin incision. Duration of labor was determined by the time lapsed in hour from mother’s complain of symptoms of true labor to skin incision. Duration of operation was determined by the time lapsed in hour from skin incision to skin closure. EBL was determined by the number of packs and gauze used during time of operation multiplied by estimated absorbable amount of blood by a single pack (1 soaked pack = 100 ml) and gauze (1 soaked gauze = 20 ml of blood) respectively. Post-operative hemoglobin represented hemoglobin level in gram/deciliter after 8 hours of post-operative period. Obesity represented maternal preoperative BMI > 25 kg/m^2^.

### Data collection tools and procedures

#### Training procedures for data collectors

Data collectors and supervisors had 1 day training before the actual data collection.

### Data collection tools and procedures for risk factors and outcome

Ethical approval to conduct this study was obtained from department of midwifery Ethical Review Committee under the delegation of Institutional Review Board (IRB) of University of Gondar. Six diploma midwives (two per shift) from obstetrics and gynecology ward were used to collect both the risk factor and outcome data. Two other Bachelor of Science (BSc) midwives from Debretabor University were assigned to supervise the data collection process. Before data collection process the data collectors informed each study participant about the purpose and anticipated benefits of the research project. The study participants had also been informed on their full right to refuse, withdraw or completely reject part or all of their part in the study. They had also been informed that their address and phone number would be used for the sole purpose of follow up and all data obtained from them would be kept confidential. Written consent was obtained from study participants prior to data collection. Study participants who were ≥ 18 years of age gave their written consent. Study participants who were < 18 years of age gave their written consent via their family members. Family members used to provide consent on the behalf of participants under the age of 18 were participants’ husbands and either of the participants’ parents. Priority was given for husbands and parents were considered in cases of either husbands’ absence or husbands’ minority age (under 18 years of age). We can confirm that the ethics committee had approved the use of attendants (husband or either of the parents) on the behalf of participants under the age of 18.

Data were collected by face to face interview of study participants, direct observation of the CS procedure and by reviewing of study participants’ charts. A semi structured and pre-tested questionnaire/data extraction tool prepared in English, translated to Amharic (local language) and then translated back to English was used to collect data. Relevant preoperative and intra operative events were observed, asked and recorded. Postoperatively, surgical wounds were examined immediately before discharge and their status recorded. Discharging study participants were counseled about symptoms of SSI and the need to return to the hospital immediately for any infected wound complaints. Their relevant addresses (phone number, place of residence and the name of the nearest health care institution) were well documented. Regular telephone calls were made at 15th and 30th post operation day [14] with an option to communicate at any time if suspicion of wound infection arose. SSI was identified before discharge and/or by readmission and evaluation of those participants with concerns of wound infection during phone calls or came directly with complain of symptoms of wound infection after discharge based on the criteria of CDC [29]**.**

### CDC criteria for defining a SSI [29]

#### Superficial incisional SSI

Infection occurs within 30 days after the operation and infection involves only skin or subcutaneous tissue of the incision and at least one of the following:

1. Purulent drainage, with or without laboratory confirmation, from the superficial incision.
2. Organisms isolated from an aseptically obtained culture of fluid or tissue from the superficial incision.
3. At least one of the following signs or symptoms of infection: pain or tenderness, localized swelling, redness, or heat and superficial incision is deliberately opened by surgeon, unless incision is culture-negative.
4. Diagnosis of superficial incisional SSI by the surgeon or attending physician.

#### Deep incisional SSI

Operation related infection involving deep soft tissues which occurs within 30 days after the operation and at least one of the following:

1. Purulent drainage from the deep incision but not from the organ/space component of the surgical site.
2. A deep incision spontaneously dehisces or is deliberately opened by a surgeon when the patient has at least one of the following signs or symptoms: fever (> 38 °C), localized pain, or tenderness, unless site is culture-negative.
3. An abscess or other evidence of infection involving the deep incision is found on direct examination, during reoperation, or by histo-pathologic or radiologic examination.
4. Diagnosis of a deep incisional SSI by a surgeon or attending physician.

### Endometritis [30]

Must meet at least one of the following criteria:

1. Patient has organism(s) identified from endometrial fluid or tissue by a culture or non-culture based microbiologic testing method which is performed for purposes of clinical diagnosis or treatment.
2. Patient has at least two of the following signs or symptoms: fever (> 38.0 °C), pain or tenderness (uterine or abdominal), or purulent drainage from uterus

### Data quality control

Proper designing and pre-testing of the questionnaires was made on 18 study participants at Debark district Hospital. Data collectors and supervisors had 1 day training before the actual data collection. Every day after data collection, questionnaires/data extraction tools were reviewed and checked for consistency and completeness by the supervisors and the necessary feedback was offered to data collectors. Any missed or incorrectly filled questionnaire was sent back to the respective data collector for correction. Epi Info version 7 was used to control Data entry errors. Data clean up and cross-checking was done before analysis.

### Data processing and analysis

All the questionnaires were checked for completeness manually and coded, then entered into Epi info 7 and exported to SPSS version 20 for further analysis. Descriptive analysis results were presented in the form of tables and text using frequencies and summary statistics such as mean, standard deviation and percentage. First Bivariate binary logistic regression analysis was done to determine the crude association of each independent variable with the outcome variable via enter method. Those crudely associated independent variables with the dependent variable using odds ratio with 95% confidence interval were entered via backward likelihood ratio for multivariate binary logistic regression analysis to adjust the influence of various independent variables (confounding effect) on the outcome variable. The Hosmer and Lemeshow (HL) test was used to check model fitness in our study. According to HL, if the significance value is more than 0.05, it indicates the model fits data well [31]. The HL test indicated that our final model was fit (*p* = 0.605). Odds ratio with 95% confidence interval were used to estimate the strength of the association between independent variables and dependent variable.

## Results

### Socio-demographic and medical characteristics

A total of 334 women were included in the study, making 96.25% response rate (Fig. 1). The age of the study participants were between 17 and 43 years with mean (±SD) age 26.38 ± 5.47 years. All of them were married and from Amhara ethnicity. About 96% of them were orthodox Christians and about 59% of them have attended secondary and above education. More than two-thirds (69%) of them were urban dwellers.

The health status of about 70% of study participants was ASA class I, about 8% of them were chronic hypertensive cases and 4.5% of them had preexisting anemia (Table 1).

**Table 1:** Socio demographic and medical characteristics of study participants of SSI and associated factors among CDs at DGH, 2017. (*n* = 334)

Variable	Frequency	Percent (%)
Age		
< 20	47	14.1
20–34	232	74.4
35–49	35	10.5
Residence		
Urban	232	69.5
Rural	102	30.5
Educational status		
Unable to read and write	56	16.8
Primary education	79	23.6
Secondary and above	199	59.6
Religion		
Orthodox	322	96.4
Muslim	12	3.6
Health status		
ASA class I	238	71.2
ASA class II	48	14.4
ASA class III	48	14.4
Chronic hypertension		
Yes	28	8.4
No	306	91.6
Diabetes mellitus		
Yes	5	1.5
No	329	98.5
Heart disease		
Yes	9	2.7
No	325	97.3
Pre-existing anemia		
Yes	15	4.5
No	319	95.5
Obesity		
Yes	14	4.2
No	320	95.8

*ASA* American society of Anesthesiologists

### Pregnancy and labor related characteristics

About 43 and 50% of participants were primigravidas and primiparas respectively with respective mean gravity and mean parity status of 2.28 times and 1.59 times. The maximum number of gravidity and parity was nine pregnancies and eight deliveries respectively. About 78% of them had term pregnancy and bout 86% of them had ANC follow up. At least one form of PIH (either gestational hypertension, pre eclampsia or eclampsia) were diagnosed in 27.1% of study participants.

On labor status of study participants, about 75% of them had labor before the operation and about 43% of them had ruptured membrane before the operation with the mean duration of 14 h and 10.51 h respectively. Eleven percent of participants had either preoperative or intraoperative chorioaminitis based on clinical findings (Table 2).

**Table 2:** Pregnancy and labor related characteristics of study participants of SSI and associated factors among CDs at DGH, 2017. (*n* = 334)

Variable	Frequency	Percent (%)
Gravidity		
1	143	42.8
2–4	150	44.9
≥ 5	41	12.3
Parity		
1	169	50.6
2–4	124	37.1
≥ 5	41	12.3
Ante natal care follow-up		
Yes	288	86.2
No	46	13.8
Pregnancy induced hypertension		
Yes	57	27.1
No	277	82.9
Gestational diabetes mellitus		
Yes	5	1.5
No	329	98.5
Gestational age		
Preterm	47	14.1
Term	260	77.8
Post term	27	8.1
Steroid therapy		
Yes	38	11.4
No	296	88.6
Labor before the operation		
No labor	84	25.1
Yes (≤24 h)	199	59.6
Yes (> 24 h)	51	15.3
Way of labor initiation		
No labor	84	25.1
Spontaneous	195	58.4
Induced	55	16.5
Vaginal examination		
No	38	11.4
Yes (≤5)	114	34.1
Yes (> 5)	182	54.5
Status of membrane		
Intact	189	56.6
Ruptured (≤ 12 h)	108	32.3
Ruptured (> 12 h)	37	11.1
Chorioaminitis		
Yes	40	12
No	294	88

### Intra operation and post operation related variables

Of the total operations about 86% of them were emergency CDs. About 31% of indications for CD was NRFHRP (Table 3), Abdomen was entered via pfannensteil incision for about 87% of operations and lower uterine segment transverse cesarean section was done for all cases. Almost all wounds were clean contaminated wounds and skin was closed via sub cuticular suture for about 93% of case. About 57% of operations were done by integrated emergency surgical officers and about 17% of participants had post operation haemoglobin value of less than 11 g/decilitre (g/dl) (Table 4).

**Table 3:** Indication for cesarean section of study participants of SSI and associated factors among CDs at DGH, 2017. (*n* = 334)

Indication	Frequency	Percent (%)
Non reassuring fetal heart rate pattern	103	30.8
Non reassuring biophysical profile	32	9.6
Cephalo-pelvic disproportion	46	13.8
Previous scar	34	10.2
Breech	23	6.9
Failed induction	17	5.1
Cervical arrest	15	4.5
Failed instrumental delivery	14	4.2
Placenta Previa	13	3.9
Twin	12	3.6
Transverse lie	7	2.1
Cord prolapse	5	1.5
Others^a^	13	3.9
Total	334	100

^a^Others: Macrosomia, Cord presentation, Triplet

**Table 4:** Intra operation and post operation related characteristics of study participants of SSI and associated factors among CDs at DGH, 2017. (*n* = 334)

Variable	Frequency	Percent (%)
Type of cesarean delivery		
Emergency	287	85.9
Elective	47	14.1
Time Prophylaxis antibiotics given		
≤ 1 h before the operation	300	89.8
> 1 h before the operation	34	10.2
Skin incision		
Pfannensteil	291	87.1
Midline	43	12.9
Procedure duration		
≤ 1 h	331	99.1
> 1 h	3	.9
Dis-impaction of head		
Yes	8	2.4
No	333	97.6
Estimated blood loss		
≤ 500 ml	328	98.2
> 500 ml	6	1.8
Intra-operation transfusion		
Yes	6	1.8
No	328	98.2
Wound class		
Clean contaminated	325	97.3
Contaminated	9	2.7
Skin closure		
Interrupted	22	6.6
Sub-cuticular	312	93.4
Qualification of surgeon		
Integrated emergency surgical officer	191	57.2
Obstetrician and gynecologist	143	42.8
Experience		
2–3 years	120	36
> 3 years	214	64
Post-operative hemoglobin		
< 11 g/dl	59	17.7
≥ 11 g/dl	275	82.3

*hr* hour, *Ml*: milliliter, *g/dl* gram/ deciliter

### Proportion of SSI

Of the total 334 study participants, 27 of them had developed SSI, making proportion of 8.1% (95%Cl: 5.4, 11.6). Three of them were identified before discharge and the remaining 24 were traced through follow up and readmission. All SSI cases were superficial incisional wound infection developed from emergency CDs.

### Factors associated with surgical site infection

In bivariate logistic regression analysis, SSI was significantly associated with ASA class III health status classification, presence of both chronic hypertension and PIH, operation after onset of labor, operation after ROM, presence of chorioaminitis, midline skin incision, interrupted skin closure, and EBL of greater than 500 ml and post-operative hemoglobin of less than 11 g/dl. Among variables found to be significantly associated with development of surgical site infection using bivariate logistic regression analysis, PIH (AOR = 4.75, 95%CI: 1.62, 13.92), chorioaminitis (AOR = 4.37, 95%CI: 1.53, 12.50), midline skin incision (AOR = 5.19, 95% CI: 1.87, 14.37 and post-operative hemoglobin less than 11 g/dl (AOR = 5.28, 95%CI: 1.97, 14.18) were also found to be significant in multivariate logistic regression analysis (Table 5).

**Table 5:** Bivariate and multivariate logistic regression analysis of Factors Associated with SSI among cesarean deliveries at DGH, 2017. (*n* = 334)

Variable	SSI		COR (95% CI)	AOR (95% CI)	*P* value
	Yes	No			
	Frequency	Frequency			
Health status based on ASA					
Class I	13	225	Reference		
Class II	6	42	2.47 (.89, 6.87)		
Class III	8	40	3.46 (1.35, 8.89)		
Chronic hypertension					
Yes	6	22	3.7 (1.35, 10.12)		
No	21	285	Reference		
PIH					
Yes	12	45	4.66 (2.05,10.60)	4.75 (1.62, 13.92)	0.000
No	15	262	Reference		
Labor status					
Yes	25	225	4.56 (1.06, 19.66)	4.53 (.95, 21.57)	
No	2	82	Reference		
Membrane status					
Ruptured	19	126	3.41 (1.45, 8.04)		
Intact	8	181	Reference		
Chorioaminitis	Frequency	Frequency			
Yes	12	28	7.97 (3,40,18.70)	4.37 (1.53,12.50)	0.000
No	15	279	Reference		
Skin incision					
Midline	12	31	7.12 (3.06, 16.58)	5.19 (1.87,14.37)	0.000
Pfannensteil	15	276	Reference		
Skin closure					
Interrupted	7	15	6.81(2.49, 18.61)		
Sub-cuticular	20	292	Reference		
EBL					
≤ 500 ml	24	304	Reference		
> 500 ml	3	3	12.67(2.42,66.18)		
Post OP Hgb					
< 11 mg/dl	15	44	7.47 (3.28, 17.02)	5.28 (1.97, 14.18)	0.000
≥ 11 mg/dl	12	263	Reference		

## Discussion

This study assessed proportion of SSI among CDs and found 8.1% [14] of CDs developed SSI (95%Cl: 5.4, 11.6).

This finding was in line with findings in Cambodia-6.25% [32], Pennsylvania-6.5% [19], Thai-Myanmar border hospital-5.9 [20], London-9.8% [33], Ankara-6.2% [12], sub Saharan Africa-7.3% [34], Egypt-9% [22], Tanzania-10.9% [8], Hawassa Teaching and Referral Hospital-11% [16], Assela Teaching and Referral Hospital-9.4% [17] and a study done in Lemlem Karl General Hospital-6.8% [18].

The result was high when compared with study done in Nanjing-3.34% [9]. This might be due a difference in quality of surgical care provision, the later assumed to have high quality service. The finding was also high when compared with another study done in southern India [35] where 4.1% of CDs had developed SSI. The possible explanation might be the magnitude represented those SSI cases developed only in first post-operative week. The result was also high when compared with a study done Israel-3.7% [13]. This difference again might reveal the difference in the quality of surgical care provisions as the later country is expected to deliver high quality service. The magnitude was again high when compared with the finding in Rwanda − 4.9% [14]. This might be due to the difference in provision of quality standard surgical care.

The magnitude of SSI was lower when compared with a study done in Nepal [10] and India [36], where 12.6, 13% and of CDs ended up with SSI respectively. These discrepancies might be tailored to differences in sample size and the difference in duration between the studies. On the other hand, SSI was diagnosed in 24.3% of Karachi [11] women following Their CD, three times higher than the finding of this study. High prevalence of obesity and diabetes mellitus among Karachi women might have contributed for such an increase SSI rate. The proportion of SSI following CDs in Nnewi (Nigeria) [21] was 12.5% that is high when compared with this study. This might be due midline skin incision was used for all previous scar cases in Nigeria which in turn is presumed to be responsible for higher rates of SSI.

The result was again low when compared with the finding of an observational study done in Tanzania [15] where 48.2% of women were found having SSI following CDS. This might be due to the difference in study setting, the later done in tertiary hospital where flow of referral cases are expected to be high.

Mothers with PIH were about five times (AOR = 4.75, 95%CI: 1.62, 13.92) more likely to develop SSI than those mothers without the problem. This finding is consistent with the findings of previous studies done in Israel [13] and Tanzania [8]. The possible explanation might be hypo perfusion of the wound caused by peripheral vasoconstriction effect of PIH. In addition those mothers with such problems might have edematous wound edges responsible for further entry of organisms and establishment of infection.

Mothers with chorioaminitis were also about four point four times (AOR = 4.37, 95%CI: 1.53, 12.50) more likely to develop SSI than those mothers without the problem. This finding is in line with the findings of the studies done in Pennsylvania [19], china [9], Thailand [20], Burkina Faso [37] and Assela [17]. This is because organisms responsible for chorioaminitis might use metritis as focus of infection and disseminate through systemic circulation to easily establish wound infection.

Operations done via midline skin incision were about five times (AOR = 5.19, 95% CI: 1.87, 14.37 more likely to develop SSI than operations done via pfannensteil incision. There are similar associations among studies done in Nepal [10], Nigeria [21], Tanzania [15], Assela [17] and Maichew [18]. The reason might be vertical incisions are more likely to be affected by intra-abdominal pressure which in turn might be responsible for poor skin integrity thereby facilitating entry of organisms and development of SSI. In addition, vertical incision has low perfusion of gases and nutrients compared with transverse incision thereby delaying would healing and increasing the likely of infection. Additional explanation might be because of prolonged duration of surgery.

Those Mothers whose post-operative hemoglobin level was less than 11 g/dl were about five point three times (AOR = 5.28, 95%CI: 1.97, 14.18) more likely to develop SSI than mothers whose hemoglobin was greater than or equal to 11 g/dl. This finding is similar with the findings of other studies done in Thailand [20], Oman [38] and Hawassa [16]. The reason behind might be due to hypo perfusion of the wound secondary to anemia and reduced post-operative ambulation.

As a limitation this study had excluded 12 study participants for the sake of tracing mechanism which might have impact on the above result. The other limitation was possible information bias as the data collectors were staffs of the same hospital. Small sample size and short period of the study were also among the limitations of this study.

## Conclusions

According to this study proportion of SSI following CDs in DGH was found to be significant in this country. PIH, chorioaminitis, midline skin incision and post-operative hemoglobin of less than 11 g/dl were independent factors associated with development of SSI. CDs with concomitant PIH, chorioaminitis and post-operative anemia needs special care and follow up until SSI is ruled out. It is also advisable to replace generous midline skin incision with pfannensteil incision.

## Data Availability

All data generated or analyzed during this study are included in this published article [and its supplementary information files].

## Abbreviations

AOR: Adjusted Odds Ratio
ASA: American Society of Anesthesiologists
BMI: Body Mass Index
CD: Cesarean Delivery
CDC: Center for Disease Control and prevention
CDs: Cesarean Deliveries
CI: Confidence Interval
COR: Crude Odds Ratio
CS: Cesarean Section
DGM: Debretabor General Hospital
DM: Diabetes Mellitus
EBL: Estimated Blood Loss
GDM: Gestational Diabetes Mellitus
IRB: Institutional Review Board
NRFHRP: Non Reassuring Fetal Heart Rate Pattern
PIH: Pregnancy Induced Hypertension
ROM: Rupture of Membrane
SPSS: Statistical Package for Social Sciences
SSI: Surgical Site Infection
